# Feasibility of genomic profiling with next-generation sequencing using specimens obtained by image-guided percutaneous needle biopsy

**DOI:** 10.1080/03009734.2019.1607635

**Published:** 2019-06-09

**Authors:** Miyuki Sone, Yasuaki Arai, Shunsuke Sugawara, Takatoshi Kubo, Chihiro Itou, Tetsuya Hasegawa, Noriyuki Umakoshi, Noboru Yamamoto, Kumiko Sunami, Nobuyoshi Hiraoka, Takashi Kubo

**Affiliations:** a Department of Diagnostic Radiology, National Cancer Center Hospital, Tokyo, Japan;; b Department of Experimental Therapeutics, National Cancer Center Hospital, Tokyo, Japan;; c Department of Pathology and Clinical Laboratories, National Cancer Center Hospital, Tokyo, Japan;; d Division of Translational Genomics, Exploratory Oncology Research & Clinical Trial Center, National Cancer Center, Tokyo, Japan

**Keywords:** Biopsy, genomic analysis, needle biopsy, next-generation sequencing

## Abstract

**Aims:** The demand for specimen collection for genomic profiling is rapidly increasing in the era of personalized medicine. Percutaneous needle biopsy is recognized as minimally invasive, but the feasibility of comprehensive genomic analysis using next-generation sequencing (NGS) is not yet clear. The purpose of this study was to evaluate the feasibility of genomic analysis using NGS with specimens obtained by image-guided percutaneous needle biopsy with 18-G needles.

**Patients and methods:** Forty-eight patients who participated in a clinical study of genomic profiling with NGS with the specimen obtained by image-guided needle biopsy were included. All biopsies were performed under local anesthesia, with imaging guidance, using an 18-G cutting needle. A retrospective chart review was performed to determine the rate of successful genomic analysis, technical success rate of biopsy procedure, adverse events, rate of success in pathological diagnosis, and cause of failed genomic analysis.

**Results:** The success rate of genomic analysis was 79.2% (38/48). The causes of failure were unprocessed for DNA extraction due to insufficient specimen volume (6/10), insufficient DNA volume (2/10), and deteriorated DNA quality (2/10). The rate of successful genomic analysis excluding NGS analysis that failed for reasons unrelated to the biopsy procedures was 95.2% (40/42). Technical success of biopsy was achieved in all patients without severe adverse events. The rate of success in the pathological diagnosis was 97.9% (47/48).

**Conclusions:** Image-guided needle biopsy specimens using an 18-G cutting needle yielded a successful NGS genomic analysis rate with no severe adverse events and could be an adoptable method for tissue sampling for NGS.

## Introduction

With the advancement of personalized cancer treatment, the demand for specimen collection for genomic profiling is rapidly increasing (1–3). In clinical practice, individualized treatments, such as molecular targeted therapies, that are selected based on the results of genomic profiling have been indicated for many types of cancer. Also, new clinical study designs, such as umbrella trials and basket trials, in which drugs are selected according to a patient’s genomic profile are increasingly conducted (4–6), and the demand for next-generation sequencing (NGS) analysis, which is capable of performing multiple genomic analyses at once, is increasing. Percutaneous needle biopsy is recognized as minimally invasive, but the tissue obtained has been considered unfit for genomic profiling because of insufficient specimen quantity compared with surgical specimens. Currently, the rate of successful genomic profiling with percutaneous needle biopsy varies from 47% to 100% (7–11). Additionally, there are only a few reports with NGS analyses using specimens obtained by percutaneous needle biopsy (10,11).

As the role of image-guided percutaneous needle biopsy for NGS genomic profiling has not been established, we aimed to evaluate the feasibility of genomic analysis using comprehensive NGS with specimens obtained by image-guided percutaneous needle biopsy with 18-G needles.

## Materials and methods

### Patients

**Figure 1.**
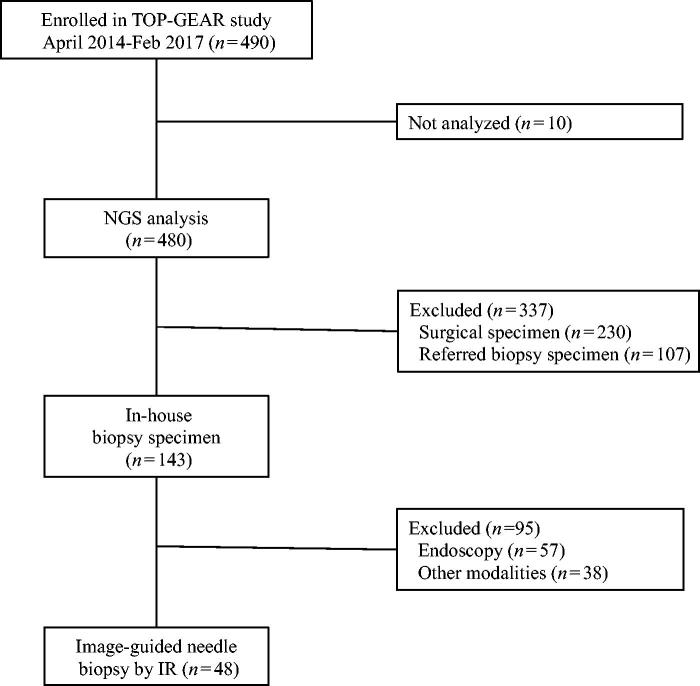
Flow chart of patient selection.

This study was a retrospective observational study employing a medical record survey. Selection criteria of the patients are the following: patients who enrolled in a clinical study of genomic profiling using a dedicated cancer gene panel for NGS (Trial of Onco-Panel for Gene-profiling to Estimate both Adverse events and Response by cancer treatment [TOP-GEAR] study: Clinical Study Registration No. UMIN000011141) between April 2014 and February 2017. We included patients undergoing genomic profiling with the specimen obtained with image-guided needle biopsy using interventional radiology technique. Patients who were evaluated with out-of-hospital/referred specimens, surgical specimens, and endoscopy specimens were excluded (Figure 1). This study is Health Insurance Portability and Accountability Act compliant, and the institutional review board at our institution waived the approval. All patients provided written informed consent for biopsy procedures.

### Biopsy procedure

**Figure 2.**
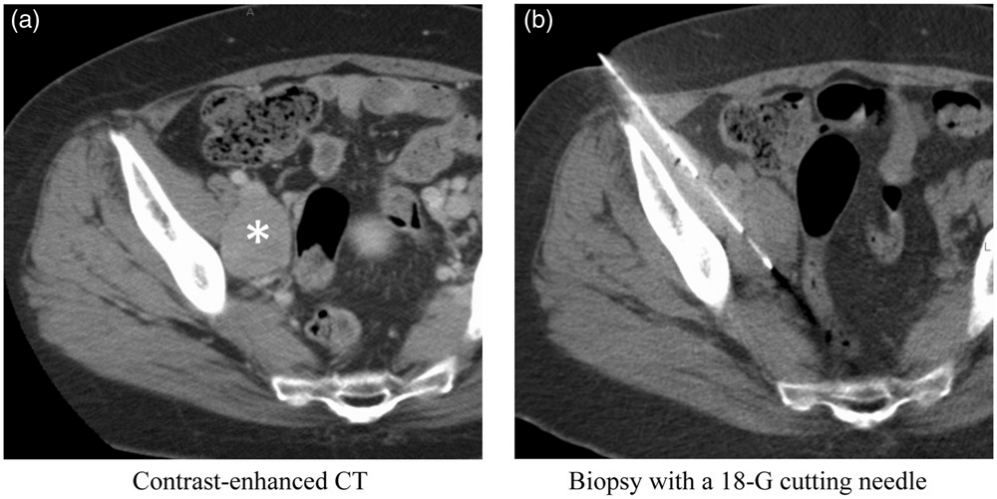
Representative case showing biopsy of a pelvic mass from a woman in her 60s. (a) Contrast-enhanced CT of the pelvis demonstrates an enhanced mass (asterisk) between the right external and internal iliac vessels. (b) Biopsy was performed using an 18-G cutting needle without penetration of the iliac arteries and veins.

Biopsy sites were selected based on the contrast enhancement on computed tomography (CT) and magnetic resonance imaging (MRI) images, MRI diffusion restriction, and increased FDG uptake on positron-emission tomography (PET) that were most likely to correspond with high viability of tumor cells. To minimize risk of complications, a puncture route that would not pass through large vessels or organs was planned (Figure 2). All biopsies were performed by board-certified interventional radiology (IR) specialists or IR fellows under supervision. Puncture was performed under local anesthesia with the guidance of angio-CT (INFX-8000C/Aquilion 16; Canon Medical Systems, Ohtawara, Japan), which is a combined CT and fluoroscopic apparatus, or ultrasonography (US) (TA 510; Canon Medical Systems, Ohtawara, Japan, or FAZONE CB; FUJIFILM, Tokyo, Japan). Images, in which the needle reached the inside of a lesion, were recorded (Figure 2). The number of specimens taken was determined according to the size of the specimens obtained and the requirement for the research. The obtained specimens were immediately fixed in 10% neutral-buffered formalin fixative and transported that day to the pathology laboratory. On-site, rapid cytology was not performed.

### Specimen processing and histopathological diagnosis

Specimens were fixed in neutral-buffered formalin for 24–72 hours and embedded in paraffin. Tissue sections of 2–3-µm thickness were prepared for hematoxylin and eosin (HE) staining, and 5–10-µm-thick sections were prepared for genomic profiling. In addition to HE staining, special stainings, immunohistochemistry, and genomic tests as insurance-approved companion diagnostics were performed as necessary, and pathological diagnosis was rendered by at least two certified pathologists. For patients who consented to the TOP-GEAR study before biopsies were taken, DNA extraction was performed immediately. In contrast, for patients who consented to and participated in the TOP-GEAR study after biopsies were taken, excess specimens harvested during a previous biopsy were used for DNA extraction.

### Genomic analysis

An NGS apparatus capable of detecting 114 cancer-related gene mutations/amplifications, 12 fusion genes, and 1 gene deletion/polymorphism in a single assay was used for NGS analysis (NCC-Oncopanel ver. 4.0) NCC-Oncopanel ver. 4.0 was developed at the authors' institution and is dedicated to the specific research of TOPGEAR described in subsection ‘Patients’. (12). Sequencing libraries were prepared using SureSelect XT reagent (Agilent Technologies, Santa Clara, CA, USA) and a KAPA Hyper Prep kit (KAPA Biosystems, Boston, MA, USA) and were analyzed on a MiSeq sequencer (Illumina, San Diego, CA, USA). Bioinformatics analysis was performed, and final decisions for the report were made in conferences by the multidisciplinary team (12).

### Outcomes ascertainment

The primary outcome was the rate of successful genomic analysis with specimens obtained by percutaneous needle biopsy. The secondary outcomes were profiling of genetic alterations, technical success rate of biopsy procedures, adverse events evaluated using the Common Terminology Criteria for Adverse Events v. 4.0, rate of success in pathological diagnosis, and cause of failed genomic analysis. Technical success of the biopsy procedure was defined as obtaining tissue sections with imaging confirmation of the biopsy needle within the target. Successful NGS analysis was defined as the ability to perform genomic analysis by NGS using DNA extracted from the specimen. The causes of failed NGS analysis were categorized as: (i) failure of the puncture of the target site (sampling error); (ii) unprocessed for DNA extraction due to insufficient specimen volume; (iii) insufficient DNA volume; and (iv) deteriorated DNA quality. We also calculated the rate of successful genomic analysis excluding NGS analysis that failed due to reasons unrelated to the biopsy procedures, i.e. reasons (ii) and (iv).

### Statistical analysis

Categorical variables were estimated as percentages and median values. In order to study whether risk factors existed for the inability to perform genomic analysis, patients were divided into two groups (patients in whom genomic analysis was possible, and patients in whom analysis was not possible), and univariate analysis was performed. Patient factors (age, sex, and primary tumor type), tumor factors (site and size), and biopsy procedure factors (needle type, number of specimens collected, and time until DNA extraction) were included as variables in the analysis. Categorical variables were analyzed using Pearson’s chi-square test, continuous variables that showed a normal distribution were analyzed by *t* test, and continuous variables that did not show a normal distribution were analyzed with the Mann–Whitney *U* test. A *P* value less than 0.05 was considered to be statistically significant.

## Results

### Patient demographics

Of the 490 patients enrolled in the TOP-GEAR study during the study period, 48 were included in this study (Figure 1). Patient demographics and tumor characteristics are given in Tables 1 and 2.

**Table 1.** Patient demographics.

	*n*	%
Gender		
Male	23	48.0
Female	25	52.0
Age, years, median (range)	54 (23–77)	
Primary site		
Lung	6	12.5
Breast	6	12.5
Unknown	6	12.5
Colon	3	6.3
Thymus	3	6.3
Bile duct	2	4.2
Pancreas	2	4.2
Others	20	41.7

**Table 2.** Characteristics of tumors.

	*n*	%
Tumor location		
Liver	20	41.7
Lung	5	10.4
Mediastinum	5	10.4
Pelvis	4	8.3
Peritoneum	3	6.3
Soft part	3	6.3
Retroperitoneum	2	4.2
Pleura	2	4.2
Superficial lymph nodes	2	4.2
Diaphragm	1	2.1
Bone	1	2.1
Tumor size, mm, median (range)	35	(11–180)
<30 mm	19	39.6
≥30 mm	29	60.4

### Feasibility of genomic analysis

**Figure 3.**
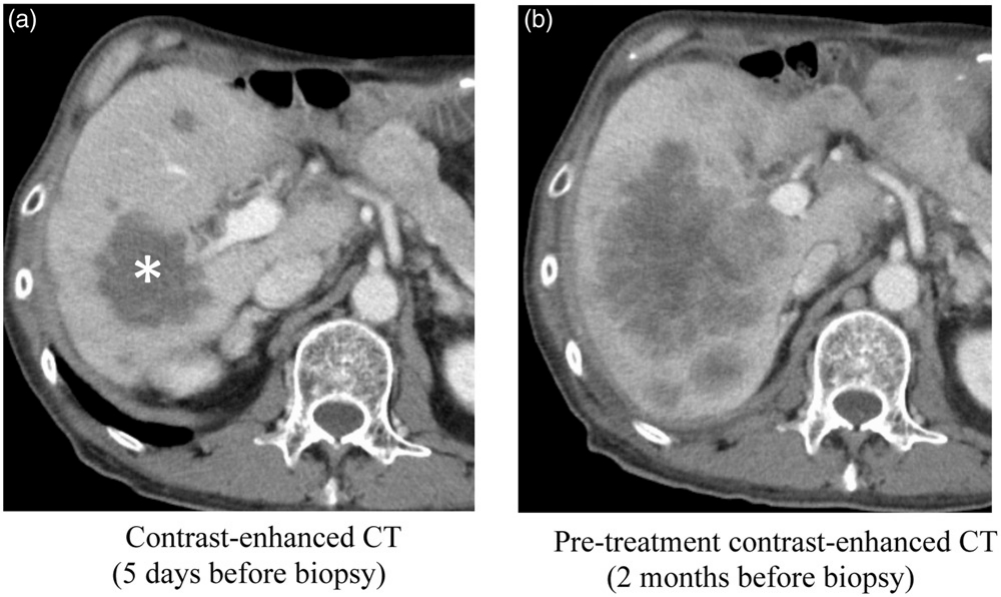
A case of failed NGS analysis. The patient is a man in his 60s with suspected liver metastases from bile duct cancer. (a) Contrast-enhanced CT 5 days before biopsy, after the anticancer medical therapy in a clinical trial. All the tumors demonstrated low attenuation representing hypovascular tumors. The largest mass in the posterior segment of the liver (asterisk) was selected for the target site of biopsy. Biopsy of five cores from various portions in the tumor was performed under ultrasound guidance. NGS analysis failed, and the pathological diagnosis was necrosis of the tumor. (b) Pre-treatment contrast-enhanced CT 2 months before biopsy. The diameters of the liver tumors are larger than that on post-treatment CT (a), and enhancement effects were seen in the periphery of the tumors.

The success rate of genomic analysis using NGS with biopsy specimens was 79.2% (38/48). A total of 52 mutations, 5 amplifications, and 2 homozygous deletions were identified. Twenty-six patients had at least one genetic alteration. The causes of failure of analysis (*n* = 10) were: (i) failure of the puncture of the target site (sampling error) (0/10); (ii) unprocessed for DNA extraction due to insufficient specimen volume (6/10); (iii) insufficient DNA volume (2/10); and (iv) deteriorated DNA quality (2/10). The rate of successful genomic analysis excluding NGS analysis that failed due to reasons unrelated to the biopsy procedures (i.e. reasons [ii] and [iv]) was 95.2% (40/42). Of the 6 specimens unprocessed for DNA extraction, 3 were due to insufficient volume of the excess specimen, and 3 were due to shortage of the specimen due to requirement of multiple immunohistochemistry tests. The imaging finding of the insufficient DNA volume of 2 patients with liver tumor was hypovascularity in the target region. In one patient, who underwent biopsy after medical treatment for liver metastases from bile duct cancer, there was a marked decrease in the enhancement and the size of the tumor on contrast-enhanced CT (Figure 3). Ages of the specimens of two patients with deteriorated DNA quality were 1001 and 1611 days, respectively.

When comparing the patients with successful and failed genomic analysis, no statistically significant differences were observed in evaluated variables (Table 3). There was a trend correlating failed genomic analysis with tumor size, although it was not statistically significant (*P* = 0.088).

**Table 3.** Comparison of patient and tumor characteristics between success and failure in gene profiling.

Age, years	Success (*n* = 38)	Failure (*n* = 10)	*P* value
Median	52	61	0.425
Gender			
Male	19	5	0.616
Female	19	6	
Tumor location			0.199
Liver	17	3	0.684
Non-liver	21	7	
Tumor size, mm			
Median	36	23	0.088
<30 mm	14	5	0.270
≥30 mm	24	5	
Type of needle			
Automatic	23	6	0.804
Semi-automatic	15	4	
Number of cores			
Median	3	3	0.171
Age of specimen, days			
Median	16	249	0.357

### Technical results of biopsies

The characteristics of the target lesions are shown in Table 3. The liver was the most common site (41.7%). The median diameter (largest length in the axial sections) of the target lesion was 35 mm, and 19 lesions (39.6%) were smaller than 30 mm. For the guiding image, US was used in 26 patients (54.2%) and angio-CT in 22 patients (45.8%). All biopsies were performed with 18-G needles with a throw length of 2 cm. Automatic biopsy needles (MUGNUM, Bard Biopsy Systems, Tempe, AZ, USA; Pro-Mag Ultra, ARGON Medical Devices, Plano, TX, USA) were used in 29 patients (60.4%) and semiautomatic biopsy needles (Temno Evolution, BD, Franklin Lakes, NY, USA; Bard Mission, Bard Biopsy Systems, Tempe, AZ, USA) in 19 patients (39.6%). The co-axial technique was used in 25 biopsies (52.1%). The median number of cores was 3 (range, 1–5). Biopsies were technically successful in all patients (100%), and the median procedure time was 20 min (range, 10–50 min).

Adverse events associated with the procedure included pneumothorax (grade 1) in 1 patient and bleeding (grade 1) in 4. Two of the bleeding patients underwent biopsy of the liver and were controlled with a needle-tract embolization. There were no severe adverse events or biopsy-related deaths.

### Pathological diagnostic yield

A histopathological diagnosis was established in 47 patients (97.9%). The diagnoses were adenocarcinoma, 21 patients; epithelioid hemangioendothelioma, 4; thymic cancer, 4; malignant mesothelioma, 2; and other, 17. The tumor cell percentage was measured in 27 patients, and the median was 60% (range, 10%–100%). Median DNA yield was 0.67 μg. The specimens were fresh (within 7 days from biopsy) in 21 patients (43.8%) and archived (8 days or more after biopsy) in 27 patients (56.2%). The median interval from biopsy to DNA analysis was 169 days (range, 10–2068 days).

## Discussion

Previous studies on genomic profiling of biopsy specimens have provided widely varying results (7–11,13–15). Lung cancer was the most commonly reported neoplasm, and *EGFR*, *KRAS*, and *ALK* analysis using polymerase chain reaction or fluorescence *in situ* hybridization was achieved in 67%–100% of the specimens (7–9,13). Few reports exist on the use of NGS in percutaneous biopsy specimens. In a retrospective study, Young et al. performed NGS analysis on formalin-fixed, paraffin-embedded (FFPE) specimens from fine-needle aspiration in patients with lung (*n* = 16) or pancreatic (*n* = 23) tumors, and genomic analysis was successful in all specimens (100%) (10). Zheng et al. performed NGS analysis on 1152 FFPE specimens from surgical, FNA, and percutaneous biopsy samples, with success rates of 99.3%, 96.9%, and 94.4%, respectively. (11). These prior retrospective studies of NGS reported higher success rates than the present study and identical to the rate without biopsy-unrelated reasons. However, details of the biopsy procedure were not described in these studies.

For genomic profiling, which requires the extraction and analysis of nucleic acids, the most important principle is to select a viable, cell-rich area on the imaging. Moreover, sites that appear enlarged in comparison to past images are considered to have higher viability (14). Intra- and inter-tumoral (with multiple organ metastases) genomic heterogeneity as well as temporary changes may affect the results of genomic analysis and the determination of a treatment plan (16,17). However, there is currently no diagnostic imaging method that indicates whether a needle biopsy specimen has been collected from a site having genetic mutations representative of the patient’s status. In the future, as radiogenomics comparing imaging data and the genomic profile of a tumor (18–20) develops, site selection may become more systematic and sophisticated.

The required specimen quantity for NGS analysis has previously been determined as a tumor cell percentage of ≥10% (21,22). If the tumor cell percentage is low, copy number validation detection is difficult, and the effects of artifacts increase (22). In our study, the median tumor cell percentage measured in 27 patients was 60%. This suggests that specimens with a high tumor cell percentage can be obtained by percutaneous needle biopsy planned and navigated by images. In a study of 1564 patients analyzed using NGS, Cho et al. reported that analysis was possible in 95.9% (1503 patients) (21), and the recommended parameters for NGS analysis were >1 mm in size and >1 unstained slide in the case of FFPE specimens. Regarding differences in the quantity of collected nucleic acid caused by needle diameter, Jamshidi et al. reported that the diameter of the needle contributes more to the quantity of collected nucleic acid than does the number of cores sampled; use of an 18-G needle yielded a 4.8–5.7-fold greater quantity of nucleic acid than use of a 20-G needle (23). In our study, use of an 18-G needle with a median of 3 cores yielded satisfactory results of genomic analysis and pathological diagnosis. Thus, if the target site is safely accessed, an 18-G needle would be suitable for acquiring samples for genomic analysis using NGS.

There are some limitations of this study. First, it was a retrospective study. Second, this study involved a small number of patients at a single facility. Third, all biopsies were obtained with an 18-G needle, so we did not compare nucleic acid yields with needle size. Fourth, surgical specimens were not examined. However, given the standardized technique of biopsy, our results at least have valuable information for the size and the number of cores of specimen.

In conclusion, taking image-guided needle biopsy specimens using an 18-G cutting needle yielded a success rate of 79.2% on genomic analysis using NGS; the rate excluding the NGS analysis that failed due to reasons unrelated to the biopsy procedures was 95.2%. Since there were no severe adverse events, we propose that image-guided needle biopsy might become an adoptable method for tissue sampling for NGS. By developing imaging-based selection methods of the target biopsy site and image-guided technology for effectively sampling the target site, the percentage of specimens that can undergo genomic analysis is expected to increase, but evaluations based on prospective studies are warranted.

## Disclosure statement

The authors declare that they have no conflict of interest.

## Funding

This work was supported by the Japan Agency for Medical Research and Development (AMED) under the Practical Research for Innovative Cancer Control Grant (16ck0106058h0003); the Ministry of Health, Labour and Welfare of Japan under Health and Labor Sciences Research Grant (H26-055); and National Cancer Center under the National Cancer Center Research and Development Fund (29-A-11).
